# Seasonal Variation of Health-Promoting Bioactives in Broccoli and Methyl-Jasmonate Pre-Harvest Treatments to Enhance Their Contents

**DOI:** 10.3390/foods9101371

**Published:** 2020-09-26

**Authors:** Vanesa Nuñez-Gómez, Nieves Baenas, Inma Navarro-González, Javier García-Alonso, Diego A. Moreno, Rocío González-Barrio, Mª Jesús Periago-Castón

**Affiliations:** 1 Department of Food Technology, Food Science and Nutrition, Faculty of Veterinary Sciences, Regional Campus of International Excellence “Campus Mare Nostrum”, Biomedical Research Institute of Murcia (IMIB-Arrixaca-UMU), University Clinical Hospital “Virgen de la Arrixaca”, University of Murcia, Espinardo, 30100 Murcia, Spain; vanesa.nunez@um.es (V.N.-G.); nieves.baenas@um.es (N.B.); inmaculada.navarro@um.es (I.N.-G.); fjgarcia@um.es (J.G.-A.); mjperi@um.es (M.J.P.-C.); 2 Phytochemistry and Healthy Foods Lab, Department of Food Science and Technology, CEBAS-CSIC, University Campus of Espinardo-25, E-30100 Murcia, Spain; dmoreno@cebas.csic.es

**Keywords:** broccoli, methyl-jasmonate, elicitation, bioactive compounds, season, dosage

## Abstract

Broccoli is a source of bioactive compounds that provide an important nutritional value. The content of these compounds can vary depending on agronomic and environmental conditions, as well as on elicitation. In this study, three crop trials were carried out to evaluate the effects of the cultivation season, the application of different dosages of methyl-jasmonate (MeJA) on the overall quality and on the total content of bioactive compounds of ‘Parthenon’ broccoli cultivated under the field conditions of southeastern Spain. Color parameters, chlorophyll content, total phenolic compounds, total flavonoids and antioxidant activity were measured to evaluate the overall quality. Moreover, individual carotenoids, phenolic compounds and glucosinolates were evaluated by high performance liquid chromatography with diode array detection (HPLC-DAD) and high performance liquid chromatography equipped with diode array detector coupled to mass spectrometer using electro spray ionization (HPLC-DAD-ESI/MS^n^). The content of total carotenoids, phenolic compounds and glucosinolates were higher in autumn compared with spring, showing increases of 2.8-fold, 2-fold and 1.2-fold, respectively. Moreover, a double application of MeJA increased the contents of total carotenoids, phenolic compounds and glucosinolates by 22%, 32% and 39%, respectively, relative to the untreated samples. Considering our results, the controlled and timely application of 250 µM MeJA to the aerial parts of the plants four days before harvest, on two consecutive days, seems to be a valid agronomic strategy to improve the health-promoting capacity of Parthenon broccoli, without compromising its overall quality.

## 1. Introduction

The *Brassica* family is a group of vegetables widely consumed around the world, including cabbages, cauliflower, Brussels sprouts, radishes and broccoli (*Brassica oleracea* L. var. *italica*) among others [1]. In the last few years, the consumption of cruciferous foods in Spain has increased. Specifically, the consumption of broccoli has undergone a significant rise, with a positive effect on the agricultural economy, particularly in the Murcia region (southeastern Spain), which is the region with the greatest production of broccoli in Europe [2,3]. This rise in consumption is related to increased adherence to healthier diets by European consumers, since this family of vegetables, and particularly broccoli, has high contents of fiber, minerals and vitamins, and is an important source of bioactive compounds with high antioxidant activity (carotenoids, phenolic compounds and glucosinolates) [4].

Although a single serving of broccoli provides a wide range of phytochemicals with beneficial effects for human health [1], the contents of these compounds vary depending on physiological, genetic and agronomic factors (including the cultivar, soil composition, agronomic treatments, climatic conditions and pre- and post-harvest treatments [5,6]). Carotenoids, one of the characteristic groups of compounds in broccoli, are natural pigments derived from the isoprenoid pathway, and are formed of a C40 backbone that differs according to the specific carotenoid being considered [7]. Carotenoid content can vary in broccoli plants as a result of environmental conditions—mainly temperature and sunlight [8,9]—while genetic factors and treatment applications can also affect the content [10]. Moreover, the content varies among the distinct parts of the plant, being higher in florets than in stalks [11]. The major carotenoids found in broccoli are β-carotene and lutein [12]. Phenolic compounds comprise one or more aromatic rings attached to hydroxyl groups [13]. Quercetin and kaempferol are the main flavonol glycosides, whereas chlorogenic and sinapic derivatives are the main hydroxycinnamic acid derivatives found in broccoli [14,15]. Glucosinolates (GLSs) are constituted by a thioglucose group, a sulphonated oxime group and a side chain derived from methionine, phenylalanine, tryptophan or a branched-chain amino acid [16]. Glucoraphanin (GRA), glucoiberin (GIB) and glucobrassicin (GBS) are the major GLSs in broccoli [17,18], and their breakdown products are important due to their health-promoting activity.

In vivo and in vitro studies have associated the consumption of broccoli and its phytochemicals with a reduction in the risk of suffering metabolic syndrome (obesity, diabetes and dyslipidaemia) and some types of cancer (lung, stomach, colon and rectal) [19,20,21]. For this reason, there is increased industrial interest in the improvement of the synthesis and accumulation of these bioactive compounds in plants, which naturally varies due to physiological, genetic and agronomic factors [6,22,23]. With regard to improving the quality of the vegetables or the content of bioactive compounds, an increase in plant stress [6,24] can lead to a higher synthesis of these secondary metabolites.

The management of agronomic and environmental conditions is very important to the content of phytochemicals. Broccoli shows little tolerance to cold and windy climates, preferring mild and bright environments with neutral soil pH [5]. Low temperatures can change the color of the florets from green to purple (anthocyanins), affecting the overall market acceptability. Elicitation is the main tool used to increase the content of secondary metabolites in vegetables, as it induces stress responses in plants. There are several types and classifications of elicitors; depending on their origin, we can differentiate among biotic, abiotic (chemical or physical) and phytohormones [23]. Another important classification is the time when the elicitor is applied; there are pre-harvest and post-harvest treatments, which can sometimes be combined. Several studies have involved the application of elicitors to broccoli plants in order to improve their nutritional properties (although this application is more common for seeds and sprouts). For instance, methionine, glucose, sucrose and mannitol applied as biotic elicitors during germination can increase the total contents of GLSs, anthocyanins and phenolics [25,26]. Ethanol and UV-C radiation have been used as abiotic, chemical and physical elicitors, respectively, and post-harvest treatments have led to an increase in GLSs and phenolic compounds [27,28]. Finally, for broccoli, phytohormones are probably the most-studied elicitor, while jasmonic acid (JA), methyl-jasmonate (MeJA) and salicylic acid are the most widely used, and have yielded increases in bioactive compounds in broccoli sprouts and adult plants [26]. Most of these studies have been performed under controlled conditions based on laboratory experiments or greenhouse control. However, as far as we know, in the scientific literature very few studies can be found under field conditions.

Taking all this into consideration, the aim of this work was to determine the effects of the application of MeJA as a pre-harvest elicitor. To achieve this goal, three consecutive crop trials were carried out to evaluate the effects of the season of application and the dosage of MeJA on the overall quality and the total content of bioactive compounds of ‘Parthenon’ broccoli cultivated under the real crop production conditions of southeastern Spain.

## 2. Materials and Methods 

### 2.1. Plant Material and Growing Conditions

Plants of the broccoli cv. Parthenon (Sakata seeds, Uchaud, France) were cultivated by the company Agrícola San Luis (La Hoya, Murcia, Spain) during the production periods of 2018 and 2019, under real field conditions. A single experiment was carried out in a 1000 m^2^ field with a plant density of 3.7 plants/m^2^. The soil in the plots had a loamy-sand texture, with a slightly alkaline pH (8.0 ± 0.4) and normal values of electrical conductivity (EC; 1.15 ± 0.24 dS/m) and organic matter content (2.10 ± 0.42%) for this type of agricultural soil in this region. The farm is located in Caniles, in Mediterranean southeastern Spain (Granada, lat. 37°26′03″ N, long. 2°43′28″ W). Parthenon is a broccoli variety with 90 days between sowing and harvest. It is very well adapted to the growing area and is one of the best-quality varieties for international trade. Weather parameters (temperature, humidity, wind, rain and sunlight exposure) during the three growing seasons of the experiments were also recorded and are presented in the Supplementary Materials (Supplementary Materials Table S1).

### 2.2. Experimental Design

**Figure 1:**
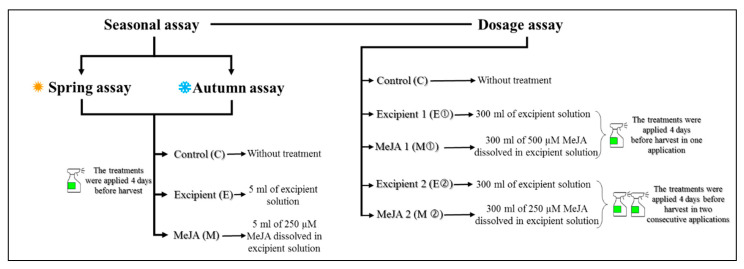
Experimental design of three assays.

Three consecutive field trials were carried out to determine the effects of the season and the dose of application of the elicitor on Parthenon broccoli, as shown in Figure 1. ‘Border’ effects were avoided by applying treatments at least 2 m away from the border. Also, the plants of the different treatments were separated by a distance of 10 m, to prevent crossing effects. Ten plants in the same development stage (commercial maturity) were treated for each treatment, with a total of 120 plants/plot in both seasonal assays and 100 in the dose of application assay.

#### 2.2.1. Seasonal Assays

The first and second assays were carried out in the same field and under the same agronomic conditions, using broccoli plants that were seeded and harvested in different seasons from two consecutive sowings: the first set of plants was grown in spring (✹) (March to early June 2018) and the second in autumn (**❄)** (mid-September to mid-December 2018). In both assays the same treatments were used. MeJA (Sigma-Aldrich, St Louis, MO, USA) was applied as a pre-harvest treatment at the same concentration. The treatments were applied to the broccoli plants four days before harvest. The samples were classified in the following experimental groups: untreated control (C); excipient (E), which was sprayed with approximately 5 mL of excipient solution (*v:v:v*) prepared with 0.5% pure ethanol, 0.5% of 20%-polyglycol (Sigma-Aldrich, St Louis, MO, USA) and 99% distilled water; and the MeJA (M) treatment, which was sprayed with approximately 5 mL of 250 µM MeJA dissolved in the excipient solution. The applications were made, using a mechanical system, to the whole aerial part of each plant for 40 plants of each group, with a total of 120 plants per assay. This dose of MeJA was selected according to the data reported previously by Ku et al. [29].

#### 2.2.2. Dose of Application Assay

In a third assay, different doses of MeJA were tested, resulting in five experimental groups according to the treatment: control (C), without any treatment; excipient 1 (E①), in which plants received one application of 300 mL of the excipient (described above) on only one day; MeJA 1 (M①), in which plants received one application of 300 mL of a solution of 500 µM MeJA; excipient 2 (E②), in which plants received two applications of 300 mL of excipient (described above), applied on two consecutive days; and MeJA 2 (M②), in which plants received two applications of 300 mL of a solution of 250 µM MeJA, applied on two consecutive days. The broccoli plants were seeded in spring and harvested in the first days of summer (mid-March to mid-June 2019). This assay was carried out in the field, and the treatment solutions were sprayed manually on the whole aerial part of the plant in the same stage of development for 20 plants of each group, with a total of 100 plants. 

For each assay, at harvest, 10 broccoli florets from each treatment were collected and analyzed. The quality parameters—including color parameters, chlorophyll content and the spectrometric assays of TPCs, TFs and ORAC—were determined for fresh samples. The bioactive compounds (carotenoids, phenolic compounds and GLS) were analyzed for freeze-dried samples, and the results were transformed to a fresh weight basis using moisture content.

### 2.3. Color Parameters

Color parameters were determined in broccoli florets with a Konica Minolta portable CR-400 chromameter (Minolta Camera Co., Osaka, Japan) (8-mm-diameter aperture, d/0 illumination system, D65 illuminant and a 2-standard observer angle), standardized using a white calibration plate. Ten broccoli florets were analyzed on the day of the harvest, upon arrival at the laboratory, recording the color parameters: lightness (L*), green-red chromaticity (a*), blue-yellow chromaticity (b*), chroma (C*) and hue angle index (h*), according to the CIELab space. For the measurements a light protection tube CR-A33e (Minolta Camera Co., Osaka, Japan) was used, taking the measures in the middle of the broccoli florets. 

### 2.4. Chlorophyll Determination

The total chlorophylls content was calculated as the sum of chlorophyll a, b and c, which were measured spectrophotometrically. One gram of fresh broccoli florets was homogenized with 10 mL of acetone/distilled water (9/1, *v/v*) in an homogenizer DI 25 basic (Ika, Germany) for 1 min at 13,500 rpm. After that, the mixture was centrifuged at 4500× *g* for 5 min, then the precipitate was washed with 10 mL of the extractant solution until the green color of the precipitate disappeared. Finally, the absorbance was measured at 665, 645 and 630 nm with a spectrophotometer (Evolution 300, Thermo Scientific, United Kingdom). The total chlorophyll content, expressed in mg/kg of fresh weight (f.w.), was obtained using the following equations [30].

Chlorophyll a = (11.6 × A_665_) − (1.31 × A_645_) − (0.14 × A_630_) (1)

Chlorophyll b = (20.7 × A_665_) − (4.34 × A_645_) − (4.42 × A_630_) (2)

Chlorophyll c = (55 × A_665_) − (4.64 × A_645_) − (16.3 × A_630_) (3)

### 2.5. Total Phenolics and Total Flavonoids Analysis

The total phenolics (TPCs) and total flavonoids (TFs) were analyzed using spectrophotometry techniques. The broccoli samples were extracted as described by Periago et al. [31], with some modifications. Briefly, 2 g of broccoli florets were extracted using a methanol/distilled water/HCl 6 N (70/29/1, *v/v*) solution. The samples were shaken in a shaking incubator (VorTemp 1550, LabNet Biotécnica, Spain) for 30 min at 37 °C, then centrifuged at 4500× *g* for 10 min. The supernatant was taken and used for the analysis of the TPCs and TFs.

The TPCs were determined using the Folin–Ciocalteu colorimetric assay as described by Singleton and Rossi [32], with some modifications. In brief, 100 μL of each extract were added to 300 μL of Folin–Ciocalteu phenol reagent diluted 1:10 (Sigma, St. Louis, MO, USA) and 400 μL of Na_2_CO_3_. The mixture was allowed to stand in darkness for 2 h, at room temperature. Gallic acid monohydrate (Sigma-Aldrich, St Louis, MO, USA) was used as the standard and the absorbance at 750 nm was measured in a spectrophotometer (Evolution 300, Thermo Scientific, Gloucester, UK). The TPC content in the broccoli samples was expressed as mg of gallic acid equivalents/kg of fresh weight (mg GAE/kg of f.w.).

The TF analysis was conducted with the colorimetric assay described by Dewanto et al. [33]. Briefly, 100 μL of each broccoli extract were mixed with 625 μL of distilled water and 375 μL of 5% NaNO_2_ solution (Merck, Darmstadt, Germany). After 6 min, 75 μL of 10% AlCl_3_6H_2_O (Probus, Barcelona, Spain) solution was added. The samples were left for 5 min, then 250 μL of 1 M NaOH (Merck, Darmstadt, Germany) were added. The absorbance was measured at 510 nm in a spectrophotometer (Evolution 300, Thermo Scientific, UK), using (+)-catechin (Sigma-Aldrich, St Louis, MO, USA) as the standard. TFs were expressed as mg of catechin equivalents/kg of fresh weight (mg CE/kg of f.w.).

### 2.6. Oxigen Radical Absorbance Capacity (ORAC)

The oxygen radical absorbance capacity (ORAC) method, described by Prior et al. [34], was used to determine the antioxidant activity of the samples, using the extract of broccoli described above for the TPC and TF analyses. This method is based on the inhibition of the oxidation induced by a peroxy-radical, using a standard with antioxidant capacity as the substrate and a fluorescent probe to measure the signal. Fluorescein was used as the indicator, Trolox as the standard and 2,2′-Azobis(2-amidino-propane) dihydrochloride (AAPH) (Sigma-Aldrich, St Louis, MO, USA) as the peroxyl radical generator. The assay was performed in a 96-well black microplate equipped with a fluorescence filter that had an excitation wavelength of 485 nm and an emission wavelength of 520 nm. For each calibration solution, the blank (0.075 M phosphate buffer, pH 7) and the samples were added to the corresponding wells. The plate reader (Synergy 2 Multi-Mode Microplate Reader, BioTek, Winooski, VT, USA) has an incubator and two injection pumps, which added the fluorescein and the AAPH during the assay; the temperature of the incubator was set to 37 °C. The fluorescence of each well was measured every 60 s for 90 min. The results are expressed as mmol of Trolox equivalents (TEs) per kg of fresh broccoli (mmol TE/kg f.w.).

### 2.7. Analysis of Carotenoids by High Performance Liquid Chromatography with Diode Array Detection (HPLC-DAD)

The analysis of carotenoids in broccoli was performed using the method described by Gonzalez-Barrio et al. [35], with some modifications. For the sample extraction, 100 mg of freeze-dried broccoli florets were extracted with 5 mL of tetrahydrofuran (THF)/methanol (50/50, *v/v*) containing 0.1% butylated hydroxytoluene (BHT), in an ultrasonic bath, for 5 min at room temperature. The extraction process was repeated, after which the residual tissue was colorless. The supernatants obtained from both extractions were mixed and dried under vacuum at 30 °C in a Laborota-4002 rotatory evaporator (Heidolph, Schwabach, Germany). The residue was re-dissolved in 2 mL of methyl tert-butyl ether (MTBE)/methanol (50/50, *v/v*) and finally centrifuged at 20,817× *g* for 5 min. This extract was analyzed by HPLC-DAD in an Agilent 1100 machine (Agilent Technologies, Las Rozas de Madrid, Spain), according to the method described by other authors [35,36,37]. A C30 column (250 × 4.6 mm, 5 μM i.d.) (Trentec, Gerlingen, Germany) was used to perform the chromatographic separation at 17 °C. The mobile phases used were MTBE (A) and methanol (B), at a flow rate of 1 mL/min. Chromatograms were recorded at 472 and 450 nm. 

Carotenoids were identified using HPLC-DAD according to their UV spectra and retention times via chromatographic comparisons with authentic standards, as well as by their spectral characteristics, based on data previously reported. Quantification was based on calibration curves constructed using 5 to 100 μg/mL lutein and β-carotene (Sigma-Aldrich, St Louis, MO, USA). The contents of total and individual carotenoids are expressed as mg/kg f.w.

### 2.8. Analysis of Glucosinolates and Phenolic Compounds by HPLC-DAD

To analyze the GLS and phenolic compounds, the multipurpose LC method described by Francisco et al. [38] was followed. Firstly, intact GLSs were identified by high performance Liquid Cromatography equipped with diode array detector coupled to mass spectrometer using electro spray ionization (HPLC-DAD-ESI-MS^n^) following their MS and MS^2^ [M-H]^−^ fragmentation ions, UV-visible spectra and order of elution, as described previously for this method [38,39]. On the other hand, phenolic compounds were tentatively identified by HPLC-DAD according to their UV-visible characteristic spectra in three different groups: caffeic acid derivatives (maximum absorption at ≈300 and ≈330 nm), flavonols (max. absorption at ≈268, ≈300 and ≈349 nm) and sinapic acid derivatives (max. absorption at 330 nm), previously described for similar LC acquisition conditions [15,38]. Both glucosinolates and phenolic compounds were quantified by HPLC-DAD in an Agilent 1200 HPLC system (Agilent Technologies, Waldbronn, Germany), using the conditions described by Baenas et al. [26]. Chromatograms were recorded at 227 nm for GLSs, at 330 nm for sinapic acids and chlorogenic acid derivatives and at 360 nm for flavonol glycosides. For the quantitative analysis of GLSs, sinigrin and glucobrassicin (Phytoplan, Germany) were used as standards for aliphatic and indole GLSs, respectively. For the phenolic compounds, chlorogenic acid, rutin and sinapic acid (Sigma, St. Louis, MO, USA) were used as the standards for the quantification of chlorogenic acid derivatives, flavonols and sinapic acids, respectively. For sample extraction, freeze-dried broccoli samples (100 mg) were extracted with 1.5 mL of methanol/Milli-Q water (70/30, *v/v*), in an ultrasonic bath, for 10 min. After that, the samples were heated at 70 °C for 30 min, shaking every 5 min, then centrifuged at 12,000× *g* for 10 min. The supernatant was dried under vacuum in a Laborota-4002 rotatory evaporator (Heidolph, Schwabach, Germany), re-dissolved in 1 mL of Milli-Q water and filtered through a 0.22-µm polyvinylidene fluoride membrane (PVDF) filter to prepare the analytical sample. The contents of total and individual GLS and phenolic compounds are expressed as mg/kg f.w.

### 2.9. Data Analysis

All analyses were performed in triplicate, with the exception of those of the color parameters, TPCs and TFs (*n* = 10), chlorophyll content (*n* = 5) and antioxidant activity (*n* = 6), and the results are expressed as the means and standard deviation (SD). An analysis of variance (ANOVA) was performed, considering the different treatments, when the data were in accordance with the assumptions of normality and homogeneity of variance. To determine differences among the mean values, a post-hoc Tukey´s test was conducted. If the data were not in accordance with normality assumptions, a Kruskal–Wallis test was selected and a Nemenyi´s test was used for post-hoc analyses. Differences were considered significant for a *p*-value < 0.05. The statistical analyses were carried out using R studio, version 3.4.3 (R Foundation for Statistical Computing, Vienna, Austria).

## 3. Results and Discussion

### 3.1. Seasonal Variation of the Quality Parameters

The same treatments were applied in both seasonal studies to florets of the Parthenon broccoli cultivar—no treatment (C), 5 mL of excipient (E) and 5 mL of 250 µM MeJA (M)—with the only differences between the two studies being the weather conditions, since they were carried out in different seasons.

#### 3.1.1. Color Parameters and Chlorophyll Content

Chlorophyll content determines the intensity of the green color of broccoli, which is important to overall quality and consumer acceptability, and hence likelihood of purchase. Table 1 shows the color parameters (L*, a*, b*, C* and h*) and the chlorophyll contents of the broccoli florets in both seasons, according to the treatment. Season had a strong and significant impact on the main color parameters and chlorophyll content, both of which affect the visual quality of broccoli. The lowest values of a* and b* were registered in the autumn samples, indicating a stronger green color and a reduced yellowing process in this season. The hue angle (h*) was significantly higher in the samples of broccoli plants cultivated in autumn than in the spring samples, showing their greener color; however, the L* value was higher in the samples harvested in spring, which could be due to the yellowing process. The yellowness of broccoli florets is a defect of the overall quality that usually appears in late spring and summer, due to the degradation of pigments at higher temperatures [40].

In the autumn assay the chlorophyll content was higher compared with the spring assay, with season having a strong effect on this parameter. These results reflect the close relationship observed between chlorophyll production and climate parameters. Our results are in accordance with those obtained by Wei et al. [40], who reported a correlation between some climate factors and the chlorophyll content of *Camellia sinensis* leaves (a negative correlation with sunlight exposure and precipitation and a positive one with daily lowest temperatures and relative humidity). For our study, we can state that the content of chlorophyll was higher in broccoli plants cultivated in autumn than in those cultivated in spring, due to the lower precipitation and shorter time of exposure to sunlight in autumn (Supplementary Materials Table S1).

The treatment with MeJA only affected the L* value and the chlorophyll content (Table 1). However, this was only observed for broccoli cultivated in autumn, since in the spring assay there were no differences among treatments in terms of total chlorophyll content, whereas in the autumn assay the treatments had a significant effect on chlorophyll content. The content of chlorophyll was highest in the control samples (C), decreasing significantly in those of groups E and M. Other authors have also reported that JA and MeJA treatments decrease the chlorophyll content in plants of the Brassicaceae family [41,42]. This occurs because JA and MeJA are involved in the senescence process, decreasing the photosynthetic electron transport rate and the total chlorophyll content and inducing the expression of several genes associated with senescence and chlorophyll catabolism [41,42]. Moreover, this effect was observed in the broccoli plants subjected to treatment E, suggesting that the ethanol and polyglycols present in the excipient produced a similar catabolic effect on chlorophyll that was more important than that of the MeJA treatment.

It is important to highlight the relationship between the color parameters and chlorophyll content. In this sense, the yellowing process that reduces the quality of the broccoli was correlated with higher values of parameter b* and lower values of h*, which also correlated with low values of chlorophyll. These results are in agreement with the chlorophyll content, which was higher in the C samples. Hence, the MeJA treatment promoted chlorophyll degradation and, consequently, the yellowing process, with differences between treated and untreated samples; however, it was only observed a decrease in L* parameter without significant change in b* and h* values. Although no changes were observed in greenness in this study, different authors [41,43] have shown a de-greening process in broccoli florets and apples when MeJA treatments are applied. The correlation between chlorophyll content and color parameters was analyzed, as shown in the Supplementary Materials (Supplementary Materials Table S2). The total chlorophyll content had a positive relationship with parameter h*, demonstrating an r value of 0.67 (*p*-value < 0.01). On the other hand, it had a negative relationship with parameter b* (*r* = −0.58, *p*-value < 0.05) in the autumn assay, a trend that was also observed in the spring, but without significance.

#### 3.1.2. Total Phenolic Compounds (TPCs), Total Flavonoids (TF) and Oxygen Radical Absorbance Capacity (ORAC)

TPCs and TF are parameters related to the radical-scavenging capacity of the plant material (i.e., the ORAC) [44]. The results obtained in these spectrophotometric assays are shown in Table 1.

There was a significant seasonal effect on these three parameters, the values of TPC, TF and ORAC being significantly higher in the broccoli cultivated in spring than in the autumn-grown plants (1.2-fold, 3.0-fold and 1.9-fold higher, respectively). These results are in agreement with those obtained by Vallejo et al. [45], who reported that all cultivars analyzed had higher values of TPCs and TFs (measured by HPLC-DAD) in spring than in winter, due to the greater sunlight exposure in spring. Moreover, Shiva et al. [46] found that the average TF contents of florets for the broccoli variety ‘NokJae’ were 3.2 mg CE/g in spring and 2.4 mg CE/g in autumn, showing the strong impact of the season. Moreover, the TPC content was higher in spring than in autumn, with means of 6.7 mg GAE/g and 4.9 mg GAE/g of broccoli florets, respectively. This behavior could be due to sunlight exposure, since a positive correlation has been described between this exposure and the biosynthesis of flavonoids and phenolic compounds [47,48]. In our seasonal assays, the sunlight exposure of Parthenon broccoli was greater in spring than in autumn.

The treatment with MeJA only had a significant effect on the content of TFs in the autumn broccoli. In the samples from plants cultivated in spring, no differences were observed among the treatments for TPCs and TFs, and no differences were found for ORAC between groups C and M; meanwhile, the application of the excipient led to a significant decrease in ORAC. These results agree with those of Kang and John [29], who did not observe differences in TPCs between untreated and MeJA-treated broccoli samples, with values of 668 and 671 mg GAE/100 g of dry weight (d.w.), respectively. Similarly, the TF content—measured as quercetin and kaempferol by HPLC—did not show differences, with the sums of both compounds being 175 and 184 µmol/100 g d.w. of broccoli in untreated and MeJA-treated samples, respectively. Moreover, Natella et al. [49] reported that the antioxidant activity of *Brotrytis cymosa* broccoli sprouts does not differ after the application of MeJA. 

Broccoli florets cultivated in autumn showed differences in TFs and ORAC, with the samples of plants treated with the excipient having higher values than those of the C and M groups. As pointed out earlier for the spring assay, in the autumn assay the TPC also did not show differences between untreated and MeJA-treated samples [29]. These results suggest that the synthesis of these secondary metabolites is affected by season, and depends on other external factors that can modify the stress metabolism of the plant more than the MeJA treatment [29].

### 3.2. Effect of Elicitor Application Dosage on the Quality Parameters

#### 3.2.1. Color Parameters and Chlorophyll Content

The color parameters and chlorophyll content in the samples of broccoli plants treated with different concentrations of MeJA are shown in Table 2. The color parameters were not affected by any of the treatments or the dose applied. By contrast, the chlorophyll content was significantly higher in C samples compared with those of the treated plants, as was observed in the seasonal assay, ranging from 362 mg/kg f.w. in C samples to 202 mg/kg f.w. in E② samples.

The excipient (ethanol and polyglycol solution) and MeJA had a catabolic effect on the total chlorophyll content in this assay, as has been described by other authors [41,42]. It should be noted that the application of neither the elicitor nor the excipient had any effect on the color parameters. Only the chlorophyll content was affected, with a significant interaction between the treatment and dosage, with the chlorophyll content decreasing at a higher dose. Moreover, this reduction did not lead to a significant change in the color parameters, suggesting that the consumer would not appreciate the chlorophyll degradation in the samples.

#### 3.2.2. TPC, TF and ORAC

According to a two-way ANOVA, the treatments influenced the parameters under analysis, with the exception of TFs (Table 2). After the application of the two consecutive doses of 250 µM MeJA (M②), a reduction in TPCs occurred (to 1993 mg/kg of f.w.), compared with the C samples (2578 mg/kg of f.w.). This agrees with the results obtained by Barrientos Carvacho et al. [50], which showed that an application of MeJA at different concentrations caused a decrease in TPCs in broccoli sprouts, the lowest concentration being obtained in the samples sprayed with 10 µM MeJA. In our study, similar results were obtained, but a significant reduction was only observed after the application of two doses of 250 µM MeJA. 

Furthermore, the ORAC values were affected by the MeJA treatment, with the samples of the treated broccoli plants (M② and M①) showing the highest values. The inverse relationship observed between the TPCs and the ORAC indicates that other antioxidant compounds such as vitamin C (which was not analyzed in the present study) could contribute to the antioxidant properties of broccoli.

### 3.3. Changes in the Contents of Carotenoids

Carotenoids were identified via HPLC-DAD according to their ultraviolet (UV) spectra and retention times, using chromatographic comparisons with authentic standards, as well as the carotenoids’ spectral characteristics, based on data previously reported [51,52,53]. This information is shown in Supplementary Materials Table S3.

**Figure 2:**
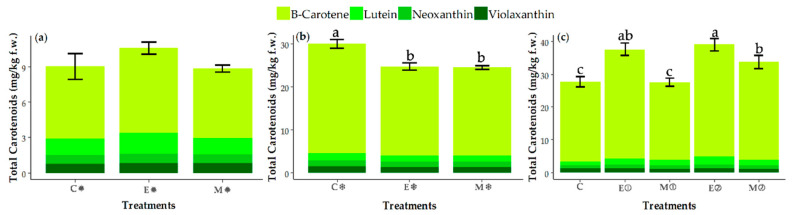
Content of individual carotenoids (β-carotene, lutein, neoxanthin and violaxanthin) (mg/kg f.w.) analyzed by HPLC-DAD, using samples of broccoli florets of the spring assay (**a**) (C✹, E✹, M✹), autumn assay (**b**) (C❄, E❄, M❄) and dosage assay (**c**) (C, E①, M①, E②, M②): C (control), E (excipient), M (MeJA), ✹ (spring), ❄ (autumn), ① (one application MeJA) and ② (two applications MeJA). Values are expressed as the mean ± SD. Different letters (**a**–**c**) indicate significant differences (*p* < 0.05) among the samples of each assay in terms of total carotenoids (*n* = 3).

The contents of carotenoids are shown in Figure 2. It can be seen that β-carotene was the main carotenoid of broccoli, representing 51% of the total carotenoids. This finding had previously been reported [12] in a study where β-carotene represented between 66% and 85% of the total carotenoids, according to the sample. In our work, the profiles and proportions of the different carotenoids differed depending on the season. In broccoli cultivated in spring, the order was β-carotene > lutein > violaxanthin > neoxanthin, while in autumn the order was β-carotene > violaxanthin > lutein > neoxanthin.

In general, the content of carotenoids was higher in broccoli cultivated in autumn than in spring-grown plants (Figure 2a,b), with season having a significant effect on the contents of individual and total carotenoids, except for lutein (Table 3). The content of total carotenoids in broccoli cultivated in autumn (26 mg/kg) represented a 2.8-fold increase compared to broccoli grown in spring (9 mg/kg). Although the effect of sunlight on the biosynthesis of *Brassica* carotenoids is not clear [10], in florets of the broccoli variety ‘Green’ the control group showed higher levels when compared with samples subjected to light of different wavelengths [54,55]. Moreover, other authors have reported that temperatures >15 °C could lead to a decrease in carotenoids [8]. Due to the higher temperature and the greater sunlight exposure during spring compared to autumn, the samples of broccoli showed different contents of total and individual carotenoids.

As can be seen in Table 3, treatments had an important effect in the season assays on all individual carotenoids and total carotenoids, except for lutein and violaxanthin. However, the application of different concentrations of MeJA did not produce a clear effect, probably due to the effect of season on the response of the plants to this elicitor. Treatment with MeJA significantly reduced the total content of carotenoids in broccoli cultivated in autumn (M**❄** group), whereas it did not show any effect on plants cultivated in spring (M**✹** group and M① group), and in some cases even led to an increase in carotenoid content (M②) (Figure 2b,c). It is notable that in the plants that received two applications of 250 µM MeJA (M②), the content of carotenoids (34 mg/kg f.w.) increased in comparison with the plants without this treatment (group C) (28 mg/kg f.w.) as well as those receiving one application of 500 µM MeJA (M①) (28 mg/kg f.w.). Regarding the effects of MeJA on carotenoid content, different results have been reported for other crop plants. Wiestra et al. [56] observed a decrease of 50% in total carotenoids content in broccoli sprouts, calculated as the sum of lutein and neoxanthin. Moreira et al. [9] described a decrease of 60% in the total carotenoids of barley leaves after treatment with MeJA. Contrastingly, Natella et al. [49] reported an increase in the β-carotene content of broccoli sprouts when MeJA was applied at a lower concentration (30 μM), whereas the β-carotene content decreased when 300-μM MeJA was applied, in comparison with untreated sprouts. These results suggest that the application of lower concentrations of MeJA in consecutive applications may be more effective for increasing carotenoid content, as we can also see in our results for Parthenon broccoli (Figure 2c).

It should be noted that chlorophyll content was directly related to the carotenoids content, with a strong correlation in the autumn assay (r value of 0.81, *p*-value < 0.01); this trend was also observed in the spring assay, but without significance (Supplementary Materials Table S2). The explanation for this relationship is that the carotenoids are the main compounds, after the chlorophylls, that take part in plant photosynthesis [57]. They absorb solar light in the spectral region not covered by chlorophylls and pass light energy to chlorophyll a, protecting it from the harmful reactions that occur in conditions of excessive light, in the presence of oxygen [57,58]. In this sense, when high temperatures reduce the content of carotenoids, as observed here in the spring assay, a reduction in the content of total chlorophylls is also observed, possibly due to the photo-oxidation process.

### 3.4. Changes in the Contents of Individual Phenolic Compounds

**Figure 3:**
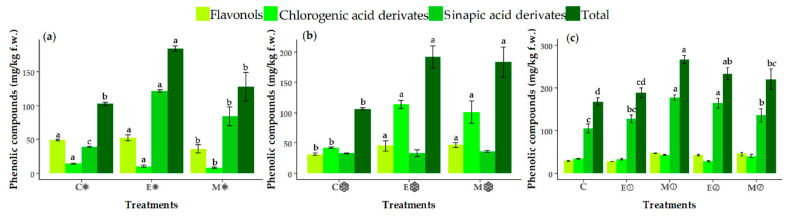
Contents of individual phenolic compounds (flavonols, chlorogenic acid derivates and sinapic acid derivates) and the total contents of phenolic compounds determined as the sums of the individual compounds (mg/kg f.w.), analyzed by HPLC-DAD for the samples of the spring assay (**a**) (C✹, E✹, M✹), autumn assay (**b**) (C❄, E❄, M❄) and dosage assay (**c**) (C, E①, M①, E②, M②): C (control), E (excipient), M (MeJA), ✹ (spring), ❄ (autumn), ① (one application MeJA) and ② (two applications MeJA). Values are expressed as the mean ± SD. Different letters (a-d) indicate significant differences (*p* < 0.05) among the samples of each assay for the individual and total phenolic compounds (*n* = 3).

Figure 3 displays the individual phenolic compounds (flavonols, chlorogenics and sinapic acid derivatives) of broccoli and the TPCs, determined as the sum of the individual compounds. There were significant effects from season, treatments and dose of MeJA on all individual phenolics and TPCs (Table 3). In general, the contents of flavonols and chlorogenic acids were higher in autumn than in spring, whereas the content of sinapic derivatives was higher in spring (Figure 3a,b). In addition to these small changes, significant differences in the content of TPCs were observed in the spring assay, with this content being higher in E✹ samples than in C✹ and M✹ samples. In the autumn assay, samples of the E**❄** and M**❄** groups had increased contents of flavonols and TPCs when compared with the C**❄** samples. The contents of individual phenolic compounds were different according to the sample, due to the interaction of season, treatment and dosage (Table 3). After the application of the different treatments, sinapic acid derivatives were more abundant than in control samples, with this trend being in the plants cultivated in spring and in the assay carried out with different dosages. By contrast, flavonols and chlorogenic acids showed a significant increase in the samples from autumn-grown plants as well as in the third assay. While some authors have reported that these three groups of phenolics are positively influenced by sunlight and UV-B exposure [45,59], others have observed that UV-A at 365 nm causes a decrease in some flavonols (mainly, quercetin glycosides and kaempferol glycosides) [60]. This influence of light on individual phenolic compounds could explain the increase in flavonols and chlorogenic acid derivates in autumn, when there is less sunlight exposure than in spring. In addition, in the third assay—also carried out in spring, with more sunlight hours—the increase in sinapic derivatives was higher than for the other individual compounds.

Although the application of MeJA altered the content of phenolic compounds, this effect was not clearly associated solely with MeJA, since broccoli plants treated with the excipient also showed a significant increase in these compounds. There was a significant effect on the content of phenolic compounds after the application of a single dose of 500 µM MeJA or two consecutive doses of 250 µM. The single dose of 500-μM MeJA caused a significant increase in TPCs, resulting in a total concentration of 266 mg/kg f.w. and representing an increase of 58% over the control, mainly due to a 69% increase in sinapic acids with respect to the C samples (Figure 3c). In the M② samples, the mean content of TPCs was 220 mg/kg of f.w., representing an increase of 32% with respect to the control samples. Different authors have shown a positive effect of the use of MeJA as a pre-harvest elicitor increasing the content of phenolic compounds in broccoli sprouts and broccoli florets [61,62]. Pérez-Balibrea et al. [61] observed that 10-μM MeJA, applied to broccoli sprouts, increased the contents of flavonoids and total phenolics by 31% and 23%, respectively, compared with higher dosages (25, 50 and 100 μM). Ku et al. [62] reported a greater accumulation of quercetin and kaempferol when 62.5- and 125-μM MeJA were applied to broccoli florets than when dosages of 250 and 500 μM were used. In the present work, the increase in TPC in broccoli samples of groups M① and M② could explain the higher ORAC values obtained, as shown in Table 2.

Moreover, we found a greater effect of the excipient and MeJA in autumn. This might be because of the greater exposure to sunlight, as other authors have reported [45], since this enhances the production of TPCs as secondary metabolites. This effect probably meant that when we added an extra stress factor—namely, MeJA—the impact was not as great as in the autumn assay, when the agronomic conditions did not stress the broccoli plants so much and thus plants were more responsive when MeJA was applied. For this reason, in the third assay, carried out in spring, the effect was observed with the higher concentration of the stressing agent, MeJA.

### 3.5. Changes in the Glucosinolate Content

The contents of GLSs in the broccoli samples of the three experimental assays are shown in Table 4. There was a clear seasonal variation, as shown in the two-way ANOVA (Table 3), with season having had a significant effect on all individual GLSs as well as their total content. Thus, the total content of GLSs was 2-fold higher in autumn than in spring. Previously, it has been reported that the total contents of GLSs were higher in spring than in autumn for two different cultivars [22], since the sunlight exposure and temperature were higher in spring. However, other authors have stated that the synthesis of GLSs is not determined only by light and temperature, as agronomic factors such as water stress can increase the GLS content [63,64]. Our results are in agreement with this, with the total precipitation in the spring assay being 361 mm compared with 185 mm in the autumn assay. As such, the water deficit in autumn could have contributed to the increase in the total GLS content. Similarly, this effect was also observed in the samples of the third assay.

Regarding the individual GLSs, the main compound in the samples of the plants cultivated in spring (in the first and third assays) was glucoiberin (GIB), followed by glucoraphanin (GRA). By contrast, the order was reversed in the broccoli cultivated in autumn, with GRA being the main compound, followed by GIB, for all treatments. Temperature is an important factor that may affect the qualitative profile of GLSs in broccoli plants. Molman et al. [65] reported that, under controlled conditions at a temperature of 18 °C, the GIB content was higher than that of GRA, while the opposite occurred at 12 °C. This explains our results, as the mean temperatures in the first and third assays were 14.8 and 16.3 °C, respectively, whereas in the second it was around 13.9 °C.

Of the two classes of GLSs present in broccoli, aliphatic GLSs were predominant in our Parthenon samples, representing on average 76%, 86% and 83%, of the total GLSs in the first, second and third assays, respectively. Chiu et al. [18] have reported that aliphatic GLSs represent around 66% of the total content of GLSs in ‘Green Magic’ broccoli.

In relation to the effect of MeJA on the content of GLSs, Chiu et al. [18] have reported that 250 μM MeJA, as a pre-harvest treatment, only increases the content of neoglucobrassicin (NGB), with an increase of 8.6-fold over the control in their study. In our study, the effect was also related to season, since 250 μM MeJA provided similar results in the spring assay, with a significant increase in NGB from 20 to 57 mg/kg f.w. Moreover, NGB was the only compound that showed a significant increase after application of MeJA in the seasonal trials, since the other GLSs decreased or did not differ with respect to the control group. The dose of MeJA applied in the third assay influenced the content of the majority of the GLSs detected (Table 3), except for GIB. It stands out that NGB increased significantly, from 0.3 mg/kg f.w., to 175 mg/kg f.w. in the broccoli treated with two consecutive doses of 250 μM MeJA, and the contents of GBSs, total indole GLSs and total GLSs also increased. In contrast, the application of one single dose of 500 μM MeJA did not enhance the contents of these compounds. Hence, the enhancement of the GLS content by MeJA applied as a pre-harvest treatment depends on the dose. In this study, the application of two consecutive doses of 250 μM MeJA had a better effect than a single dose of 500 μM MeJA, which is disagreement with the data reported by other authors, who recommended a treatment with 500 μM MeJA [62]. This enhancement is linked to season, since MeJA had no effect on the GLS content in the samples of broccoli plants cultivated in autumn.

## 4. Conclusions

Considering that broccoli is a rich source of bioactive compounds, our results are of great interest to growers and food-trading industries, particularly in Murcia (Spain), the major broccoli-growing region in Europe. New strategies need to be implemented in the field to seek out the best agronomic practices and conditions by which to improve the health-promoting capacity of broccoli, without compromising its overall quality, although the effects of MeJA could be modified by seasonal conditions and other agronomic parameters. From this study we can conclude that the controlled and timely (four days before harvest) application of 250 µM MeJA as an elicitor to the aerial parts of the plants, on two consecutive days, yielded florets of Parthenon broccoli with higher contents of bioactive compounds, without changing its overall quality.

## Figures and Tables

**Table 1:** Color parameters, chlorophyll content (mg/kg of f.w.), total phenolic compounds (TPCs) (mg GAE/kg f.w.), total flavonoids (TFs) (mg CE/kg f.w.), and oxygen radical absorbance capacity (ORAC) (mmol TE/kg f.w.) in broccoli cultivated during the spring (✹) and autumn (❄) season trials, according to the control (C), excipient (E) and MeJA (M) experimental groups.

Season	Treatment	L*	a*	b*	C*	h*	Total Chlorophylls	TPCs	TF	ORAC
Spring	C✹	43 ± 2 ^a^	−8 ± 2	13 ± 4	15 ± 4	122 ± 7	149 ± 17	1081 ± 147	207 ± 37	31 ± 9 ^ab^
	E✹	42 ± 2 ^ab^	−7 ± 2	14 ± 4	15. ± 4	116 ± 7	152 ± 38	932 ± 29	187 ± 24	20 ± 8 ^b^
	M✹	41 ± 2 ^b^	−5 ± 4	15 ± 3	16 ± 3	108 ± 12	128 ± 27	1102 ± 136	180 ± 13	44 ± 0 ^a^
Autumn	C❄	38 ± 2 ^b^	−10 ± 1	10 ± 2	14 ± 2 ^b^	135 ± 3	264 ± 25 ^a^	828 ± 86	74 ± 7 ^b^	18 ± 4 ^ab^
	E❄	41 ± 2 ^a^	−11 ± 2	14 ± 4	18 ± 4 ^a^	129 ± 4	147 ± 32 ^c^	912 ± 113	92 ± 15 ^a^	22 ± 2 ^a^
	M❄	37 ± 1 ^b^	−10 ± 1	12 ± 2	16 ± 2 ^ab^	132 ± 3	202 ± 29 ^b^	870 ± 108	26 ± 8 ^c^	14 ± 6 ^b^
ANOVA	SEASON	***	***	*	ns	***	***	***	***	***
	TREATMENT	***	ns	ns	ns	ns	***	ns	***	**
	T × S	**	*	ns	ns	*	***	**	***	***

Values followed by different letters (a–c) within each column, for each season, are significantly different according to Tukey’s test (*p* < 0.05; ns: not significant; *, ** and *** significant at *p* < 0.05, 0.01 and 0.001, respectively). Mean values ± SD (*n* = 10 for color, TPC (total phenolic compounds) and TF (total flavonoids) analyses, *n* = 5 for chlorophyll content and *n* = 6 for ORAC (oxygen radical absorbance capacity) analysis).

**Table 2:** Color parameters, chlorophyll content (mg/kg of f.w.), total phenolic compounds (TPCs) (mg GAE/kg f.w.), total flavonoids (TFs) (mg CE/kg f.w.), and oxygen radical absorbance capacity (ORAC) (mmol TE/kg f.w.) of broccoli samples in the dose of application assay for the five experimental groups: control (C), excipient (E① and E②) and one application of MeJA (M①) or two applications of MeJA (M②).

Sample	L*	a*	b*	C*	h*	Total Chlorophylls	TPCs	TF	ORAC
C	20 ± 14	−4 ± 3	5 ± 2	7 ± 3	121 ± 14	362 ± 32 ^a^	2578 ± 157 ^a^	409 ± 44	66 ± 7 ^b^
E①	17 ± 12	−2 ± 3	6 ± 3	6 ± 4	102 ± 20	276 ± 27 ^b^	2531 ± 4 ^ab^	405 ± 48	75 ± 5 ^ab^
M①	16 ± 6	−4 ± 0	9 ± 0	10 ± 0	114 ± 2	205 ± 28 ^b^	2038 ± 179 ^ab^	444 ± 55	79 ± 6 ^a^
E②	18 ± 11	−2 ± 2	8 ± 4	8 ± 4	103 ± 11	202 ± 21 ^b^	2342 ± 295 ^ab^	477 ±53	69 ± 6 ^b^
M②	27 ± 23	−6 ± 7	15 ± 9	16 ± 11	107 ± 11	243 ± 30 ^b^	1994 ± 261 ^b^	446 ± 52	83 ± 3 ^a^
TREATMENT	ns	ns	ns	ns	ns	ns	**	ns	***
DOSAGE	ns	ns	ns	ns	ns	ns	ns	ns	ns
T × D	ns	ns	ns	ns	ns	**	ns	ns	*

Values followed by different letters (a–b) within each column are significantly different according to Tukey’s test (*p* < 0.05; ns: not significant; *, ** and *** significant at *p* < 0.05, 0.01 and 0.001, respectively). Mean values ± SD (*n* = 10 for color, TPC (total phenolic compounds) and TF (total flavonoids) analyses, *n* = 5 for chlorophyll content and *n* = 6 for antioxidant activity).

**Table 3:** Results of two-way ANOVA of carotenoids, phenolic compounds and glucosinolates (GIB: glucoiberin (3-methylsulfinylpropyl-gls), GRA: glucoraphanin (4-Methylsulphinylbutyl-gls), HGB: hydroxyglucobrassicin (4-Hydroxy-3-indolylmethyl-gls), GBS: glucobrassicin (3-Indolylmethyl-gls), MGB: 4-Methoxyglucobrassicin (4-Methoxy-3-indolylmethyl-gls), NGB: neoglucobrassicin (1-Methoxy-3-indolylmethyl-gls), Total GLS: total glucosinolates) from three seasonal and dosage assays.

ANOVA	B-Carotene	Lutein	Neoxanthin	Violaxanthin	Total Carotenoids	Flavonols	Chlorogenic Acid Derivatives	Sinapic Acid Derivatives	Total Phenolics	GIB	GRA	HGB	GBS	MGB	NGB	Aliphatics	Indoles	Total GLS
**SEASON**	***	ns	***	***	***	***	***	***	**	**	***	***	***	**	***	***	***	***
**TREATMENT**	***	ns	**	ns	***	***	*	***	***	ns	*	***	**	ns	*	ns	*	ns
**T × S**	***	ns	***	*	***	***	**	***	*	ns	**	***	ns	*	***	ns	**	ns
**TREATMENT**	***	***	*	**	***	***	***	***	***	ns	***	−	*	***	***	***	***	***
**DOSAGE**	**	*	ns	ns	**	***	**	*	*	ns	***	−	**	***	***	ns	***	***
**T × D**	*	ns	ns	ns	*	***	Ns	***	***	ns	***	-	***	***	***	**	***	***

ns: not significant; *, ** and *** significant at 0.05, 0.01 and 0.001, respectively.

**Table 4:** Content of glucosinolates (mg/kg f.w.) in broccoli florets analyzed by HPLC-DAD for both seasonal assays (spring and autumn) and the dosage application assay. Glucosinolates are presented as individual compounds (GIB: glucoiberin (3-Methylsulfinylpropyl-gls), GRA: glucoraphanin (4-Methylsulphinylbutyl-gls), HGB: hydroxyglucobrassicin (4-Hydroxy-3-indolylmethyl-gls), GBS: glucobrassicin (3-Indolylmethyl-gls), MGB: 4-Methoxyglucobrassicin (4-Methoxy-3-indolylmethyl-gls), NGB: neoglucobrassicin (1-Methoxy-3-indolylmethyl-gls)), grouped as aliphatic and indole glucosinolates, and as total glucosinolates, calculated as the sum of the different individual compounds.

Assay	Sample	Glucosinolates								
		GIB	GRA	HGB	GBS	MGB	NGB	Total Aliphatic	Total Indole	Total
Spring	C✹	198 ± 9 ^a^	48 ± 20 ^b^	3 ± 1 ^b^	23 ± 1	19 ± 1	20 ± 0 ^b^	245 ± 16	65 ± 2	310 ± 15
	E✹	127 ± 8 ^b^	124 ± 35 ^a^	10 ± 1 ^a^	23 ± 2	25 ± 1	12 ± 0 ^b^	251 ± 27	70 ± 4	322 ± 23
	M✹	150 ± 34 ^ab^	106 ± 9 ^ab^	4 ± 1 ^b^	16 ± 5	19 ± 5	57 ± 15 ^a^	255 ± 43	96 ± 26	351 ± 69
Autumn	C❄	254 ± 63	300 ± 9 ^a^	2 ± 1	119 ± 12 ^ab^	39 ± 10	53 ± 13 ^b^	554 ± 64	212 ± 16 ^a^	766 ± 77
	E❄	227 ± 3	300 ± 18 ^a^	1 ± 1	132 ± 1 ^a^	27 ± 2	71 ± 1 ^a^	527 ± 20	232 ± 2 ^a^	759 ± 20
	M❄	219 ± 12	258 ± 19 ^b^	1 ± 0	107 ± 7 ^b^	27 ± 2	48 ± 5 ^b^	477 ± 32	183 ± 12 ^b^	661 ± 43
Dosage Application	C	153 ± 8	216 ± 16 ^a^	tr	8 ± 1 ^c^	*tr* ^b^	0 ± 1 ^c^	369 ± 24 ^a^	8 ± 2 ^c^	377 ± 25 ^b^
	E①	169 ± 6	137 ± 3 ^b^	tr	14 ± 0 ^b^	1 ± 0 ^b^	19 ± 1 ^b^	306 ± 9 ^b^	34 ± 2 ^b^	340 ± 11 ^b^
	M①	153 ± 50	82 ± 13 ^d^	tr	tr ^d^	*tr* ^b^	25 ± 8 ^b^	236 ± 40 ^c^	25 ± 8 ^b^	261 ± 32 ^c^
	E②	171 ± 7	103 ± 4 ^cd^	tr	1 ± 2 ^d^	1 ± 1 ^b^	6 ± 3 ^c^	273 ± 11 ^bc^	84 ^c^	281 ± 14 ^c^
	M②	186 ± 8	106 ± 5 ^c^	tr	21 ± 4 ^a^	37 ± 2 ^a^	175 ± 6 ^a^	291 ± 13 ^bc^	233 ± 12 ^a^	524 ± 25 ^a^

Values followed by different letters (a–d) within each column, for each assay, are significantly different according to Tukey′s test (*p* < 0.05). Mean values ± SD (*n* = 3). tr: traces. C (control), E (excipient), M (MeJA), ✹ (spring), ❄ (autumn), ① (one application MeJA) and ② (two application MeJA).

## Supplementary Materials

The following are available online at https://www.mdpi.com/2304-8158/9/10/1371/s1, Table S1: Temperature (°C), humidity (%), wind (km/h), rain (mm) and sunlight exposure (h/day) during the three growing seasons; Table S2: Pearson´s correlation factor for the relationship between the chlorophyll content and the color parameters (L*, a*, b*, C* amd h*) and the total carotenoids content in the three assays; Table S3: Tentative identification of individual carotenoids of broccoli florets analysed by HPLC-DAD.
