# Supplementary material for: Seasonal Variation of Health-Promoting Bioactives in Broccoli and Methyl-Jasmonate Pre-Harvest Treatments to Enhance Their Contents
Source: Foods. 2020 Sep 26;9(10):1371. doi: 10.3390/foods9101371 (PMC7599945; doi:10.3390/foods9101371)
Supplement: Supplementary file 1 [file foods-09-01371-s001.pdf]

Table S1. Temperature (°C), humidity (%), wind (km/h), rain (mm) and sunlight exposure (h/day) during the three growing seasons.

	Temperature[1]			Humidity [1]			Wind [1]		Rain [1]		Sunlight Exposure [1]
	Min	Max	Mean	Min	Max	Mean	Max	Mean	Total	Days	Hours/Day
**Spring**	9.1	21.2	14.8	43.5	86.8	67	39.8	2.6	361.2	51	13.75
**Autumn**	8.8	21	13.9	47.5	87.3	72	34.5	2.8	185	30	10.8
**Dosage-application**	9	23.9	16.3	31.5	79.5	56.8	32.5	2.8	123.4	20	13.75

The accumulated weather variables were calculated based on the number of days from sowing to harvest.

Table S2. Pearson´s correlation factor for the relationship between the chlorophyll content and the colour parameters (L*, a*, b*, C* amd h*) and the total carotenoids content in the three assays.

	Assay	L*	a*	b*	C*	h*	Total Carotenoids
**Total chlorophylls**	**Spring** ✷	−0.04	−0.01	−0.31	0.34	0.14	0.52
**Total chlorophylls**	**Autumn** ❄	−0.36	0.56 (*)	−0.58 (*)	−0.57 (*)	0.67 (**)	0.81 (**)
**Total chlorophylls**	**Dosage**	0.04	−0.04	−0.27	−0.25	0.16	−0.35

The correlation is significant at p-value<0.05 (*) or at p-value<0.01(**).

Table S3. Tentative identification of individual carotenoids of broccoli florets analysed by HPLC-DAD.

Retention Time (min)	λ_{max} (nm)	Tentative Identification	Method of Identification[1]
5.3	414, 438, 468	Neoxanthin	B, C
6.4	416, 438, 466	Violaxanthin	B, C
9.5	418, 442, 472	Lutein	A, B, C
31.8	430, 450, 476	β-carotene	A, B, C

[1]Method of tentative identification: (A) by comparison with the retentions times and wavelengths of maximum absorption in the UV/Vis spectra of authentic standards; (B) by spectral interpretation of the wavelengths of maximum absorption in the UV/Vis spectra and by comparison with the wavelengths of maximum absorption in the UV/Vis spectra reported by the bibliography [51–53]; (C) by comparison the order of chromatographic elution reported by the bibliography [52, 53].
